# A *Drosophila* XPD model links cell cycle coordination with neuro-development and suggests links to cancer

**DOI:** 10.1242/dmm.016907

**Published:** 2014-11-27

**Authors:** Karin Stettler, Xiaoming Li, Björn Sandrock, Sophie Braga-Lagache, Manfred Heller, Lutz Dümbgen, Beat Suter

**Affiliations:** 1 Institute of Cell Biology, University of Bern, 3012 Bern, Switzerland.; 2 Department of Biology, Philipps-University Marburg, 35032 Marburg, Germany.; 3 Department of Clinical Research, University of Bern, 3010 Bern, Switzerland.; 4 Institute of Mathematical Statistics and Actuarial Science, University of Bern, 3012 Bern, Switzerland.

**Keywords:** Xeroderma pigmentosum, Cell cycle synchronization, Xpd, Mitotic defect

## Abstract

XPD functions in transcription, DNA repair and in cell cycle control. Mutations in human *XPD* (also known as *ERCC2*) mainly cause three clinical phenotypes: xeroderma pigmentosum (XP), Cockayne syndrome (XP/CS) and trichothiodystrophy (TTD), and only XP patients have a high predisposition to developing cancer. Hence, we developed a fly model to obtain novel insights into the defects caused by individual hypomorphic alleles identified in human XP-D patients. This model revealed that the mutations that displayed the greatest *in vivo* UV sensitivity in *Drosophila* did not correlate with those that led to tumor formation in humans. Immunoprecipitations followed by targeted quantitative MS/MS analysis showed how different *xpd* mutations affected the formation or stability of different transcription factor IIH (TFIIH) subcomplexes. The XP mutants most clearly linked to high cancer risk, Xpd R683W and R601L, showed a reduced interaction with the core TFIIH and also an abnormal interaction with the Cdk-activating kinase (CAK) complex. Interestingly, these two XP alleles additionally displayed high levels of chromatin loss and free centrosomes during the rapid nuclear division phase of the *Drosophila* embryo. Finally, the *xpd* mutations showing defects in the coordination of cell cycle timing during the *Drosophila* embryonic divisions correlated with those human mutations that cause the neurodevelopmental abnormalities and developmental growth defects observed in XP/CS and TTD patients.

## INTRODUCTION

**Fig. 1.**
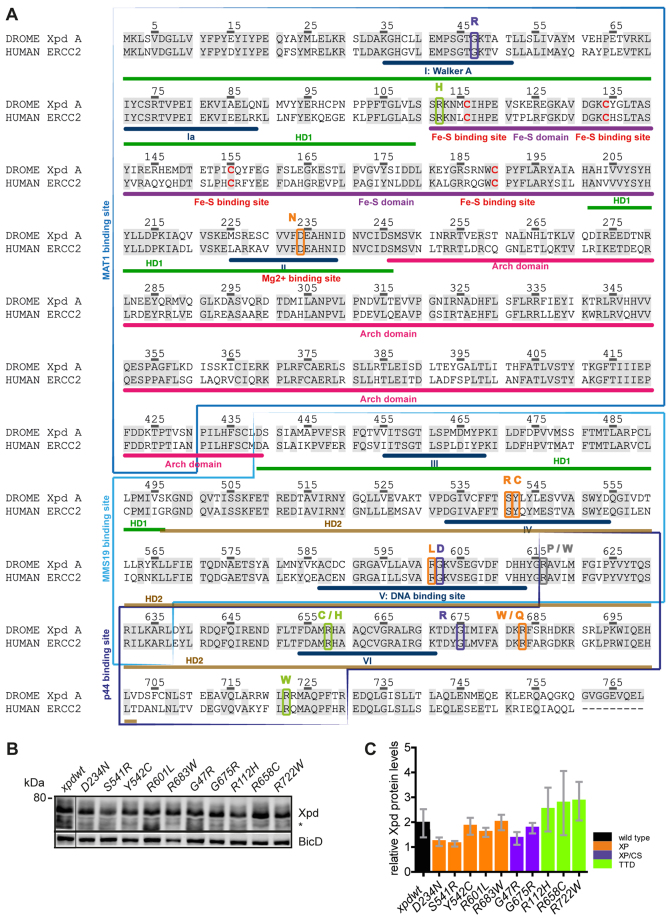
**Human and *Drosophila* Xpd: motifs, interaction sites, mutations and protein expression.** (A) Protein sequence alignment of the human ERCC2 protein (UniProt code P18074) and the *Drosophila* Xpd isoform A (UniProt code Q7KVP9) using ClustalW. Conserved residues are shaded in grey. Note that the positions are equivalent in the two sequences. Domains are indicated and labeled underneath the sequence according to Fan et al. (Fan et al., 2008). The SF2 helicase motifs are also numbered in Roman numerals. *xpd* mutations created in *Drosophila* are surrounded with a colored box with the amino acid change indicated on top. XP mutations are boxed in orange, XP/CS mutations in purple, TTD mutations in green and lethal human mutations in grey. Interaction regions mapped by point and deletion mutants are surrounded by blue boxes (Sandrock and Egly, 2001; Dubaele et al., 2003; Ito et al., 2010). (B) Representative western blot of whole-fly extracts from 1- to 3-day-old male flies to determine Xpd protein levels from the different *xpd* mutants. The polyclonal antibody used was raised against most of the Xpd open reading frame (to the Bam H1 site; Chen et al., 2003; Li et al., 2010). The unrelated BicD protein was used as a loading standard. Genotype of the flies*: w; xpd^P^; xpd^mutants^, G147*. *non-specific cross-reactive band. (C) The mean±s.e.m. Xpd protein levels for the different *xpd* mutants are shown. Results are calculated from three independent experiments.

Xeroderma pigmentosum (XP) is a rare recessive genetic disorder in humans that causes hyperpigmentation of the skin under sun exposure and cutaneous abnormalities. Among eight XP causative genes, *XPD* (also known as *ERCC2*) and *XPB* (also known as *ERCC3*) encode two helicases of the transcription factor IIH (TFIIH) complex, which functions in transcription and nucleotide excision repair (NER), and one of them, *XPD*, also functions in cell cycle regulation and chromosome segregation (Cameroni et al., 2010; Egly and Coin, 2011). Mutations in the human *XPD* gene can also cause trichothiodystrophy (TTD) and occasionally Cockayne syndrome (CS), which is seen only in combination with XP (XP/CS) in XP-D patients (Fig. 1A; supplementary material Table S1; Fan et al., 2008; Lehmann, 2001). XP mutations cause a 2000–10,000-fold increase in skin cancer risk, and some XP patients also display primary neuronal degeneration (Bradford et al., 2011). TTD patients display developmental retardation, neurological abnormalities due to dysmyelination, and some also have photosensitivity, but they do seem not to be predisposed to developing skin cancer (Hashimoto and Egly, 2009; Bergmann and Egly, 2001). TTD mutations seem to primarily cause a defect in the transcription function of TFIIH, and the severity of the transcriptional defects parallels the severity of the clinical phenotype of TTD patients (Berneburg and Lehmann, 2001). CS seems to be caused by a defect in transcription-coupled NER (Marteijn et al., 2014), causing transcriptional stress, accelerated aging and cell death. CS also causes severe sun sensitivity, premature aging and severe developmental defects such as reduced body size, skeletal abnormalities and eye problems (Kraemer et al., 2007; Laugel, 2013). Furthermore, XP-D mutations of the combined XP/CS type have been described to uncouple the incision process from the DNA damage (Berneburg et al., 2000) and to cause UV-induced strand displacement (Godon et al., 2012).

DNA is under constant attack and needs to be repaired, in particular when exposed to mutagenic agents. NER is one of the pathways that repairs helix-distorting lesions, which can be induced by chemical agents or UV, and it depends on TFIIH components, including XPD (Lehmann, 2003). The role of XPD in NER is well established; it acts as a component of TFIIH and unwinds the DNA at the site of damage (Coin et al., 2007). Its ATPase and helicase activities are crucial for this function, and through this pathway XPD seems to play important roles in surviving mutagenic attacks and in preventing premature aging.

The cyclin-dependent kinase (Cdk)-activating kinase (CAK) complex consists of Cdk7, Mat1 and cyclin H. It is present as a subcomplex of TFIIH or as free trimeric CAK. Although the free form activates the cell cycle Cdks, the TFIIH-associated CAK complex seems to primarily act in transcription by phosphorylating RNA polymerase II at its C-terminal domain (Rossignol et al., 1997; Yankulov and Bentley, 1997; Larochelle et al., 2006). Because it associates with CAK and core TFIIH (cTFIIH), XPD can act as bridge between the CAK complex and TFIIH and thereby regulate the different activities of CAK (Roy et al., 1994; Drapkin et al., 1996; Sandrock and Egly, 2001; Chen et al., 2003; Li et al., 2010). *Drosophila* embryos lacking Xpd display a high frequency of cell cycle problems associated with severe chromosomal instability during mitosis. It is therefore conceivable that reduced *XPD* function during human mitosis causes also cell cycle problems that lead to chromosomal instability. To better understand how the *XPD* mutations affect the different TFIIH complexes, it is important to measure the interaction between the mutant XPD and its TFIIH partners. In order to recruit the CAK complex to TFIIH, the N-terminal region of Xpd (Fig. 1A) binds to the Mat1 subunit of CAK and the C-terminal region binds to the p44/SSL1 subunit of cTFIIH, comprising the subunits XPB/SSL2 (known as Hay in *Drosophila*), p52/TFB2 (known as Mrn in *Drosophila*), p62/TFB1, p34/TFB4 and p8/TTDA/TFB5 (see Fig. 3A).

**TRANSLATIONAL IMPACT**

**Clinical issue**

Xeroderma pigmentosum (XP) is a rare recessive genetic disorder in humans that causes hyperpigmentation of the skin under sun exposure, cutaneous abnormalities and a 2000–10,000-fold increase in skin cancer risk. Individuals with XP also display primary neuronal degeneration. One of the causative genes of XP is *XPD* (also known as *ERCC2*). Mutations in the human *XPD* gene can also cause trichothiodystrophy (TTD) and occasionally Cockayne syndrome in conjunction with XP (XP/CS). Individuals with TTD display developmental retardation and neurological abnormalities whereas CS causes transcriptional stress, leading to severe sun sensitivity, premature aging and cell death. Additionally, CS also leads to severe developmental defects such as reduced body size, skeletal abnormalities and eye problems. Xpd is a multitasking protein that plays essential roles in cell cycle regulation, transcription and DNA repair. At the molecular level, it plays both a structural and an enzymatic role, and it is found in at least four different protein complexes. Despite much work in this area, the causal relationship between Xpd mutations, its molecular and cellular activity, and the organismal phenotype remains unclear. At present, it is therefore not possible to predict the clinical outcome of combinations of human *XPD* mutant alleles. Only very few multicellular models have been developed and the contribution of individual alleles to the different patient phenotypes could often not be established firmly.

**Results**

This study describes a novel *Drosophila* model that allows detailed biochemical and cellular studies of the *XPD* mutant alleles. The use of this model revealed interesting links to different features of the human syndromes. The activity of mutant Xpd in coordinating cell cycle timing during the *Drosophila* syncytial divisions correlated with the neurodevelopmental abnormalities and developmental growth defects observed in individuals with XP/CS and with TTD. Altered interactions of the mutant Xpd with its molecular partners in larger protein complexes appear to be a risk factor for cancer susceptibility if these defects occur together with increased mitotic defects that lead to chromosomal instability.

**Implications and future directions**

This fly model provides novel insights into the defects caused by individual hypomorphic alleles identified in individuals with XP-D. In particular, it provides a tool for researchers to assess the activity of an individual mutant variant in the various processes in which Xpd is involved. This model not only represents a good system to study the effect of specific single alleles, but could also allow the modeling of any combination of two alleles as found in human patients. This might in turn allow researchers to investigate how these combinations perform *in vivo* and which of them lead to phenotypes as found in humans.

We set out to assess *in vivo* the non-transcription functions of the different hypomorphic alleles identified from human XP-D patients. For this, we produced transgenic *Drosophila* lines that carry, as the sole source of Xpd, mutant alleles with the substitutions identified in human XP-D patients. Determining their UV sensitivity, we found that Xpd D234N (XP), G47R and G675R (XP/CS) showed the strongest lethality upon UV irradiation. Assessing the cell cycle control function of the mutant Xpd revealed a strong correlation between mutations that caused defects in the synchronization of the mitotic cell cycles and those that cause the neurodevelopmental and growth defects observed in XP/CS and TTD patients. The effect of the mutations on TFIIH complex stability was assessed in two complementary assays, which showed that the two XP mutations most clearly linked to cancer, R683W and R601L, affected the interaction of Xpd with cTFIIH and CAK. Interestingly, these two mutants also showed a high frequency of mitotic defects and chromosomal instability.

## RESULTS

### Modeling human *XPD* alleles in *Drosophila*

The *Drosophila xpd* alleles were engineered such that the transgenes were under the control of the endogenous promoter and *xpd* was either wild type or contained the same mutation as reported for the human patients. All transgenes were inserted into the 64A landing platform (Bischof et al., 2007) and then crossed into *xpd^P^* (a null *xpd* mutation) flies (Li et al., 2010) that also expressed the *jupiter* gene trap line *G147*, which marks microtubules with GFP in a way that does not affect microtubule function (Morin et al., 2001). The use of the landing platform technology in combination with the *ΦC31* integration system allowed us to insert all the different transgenes into the exact same position in the genome, thereby reducing position effect influences and allowing us to directly compare the phenotypes of individual lines. In this way, we constructed embryos and flies that solely expressed one of the 15 mutant alleles or the wild-type *xpd* allele (*xpd^wt^*) from the transgene (Fig. 1; supplementary material Table S1). We established such transgenic lines for the *xpd^wt^*, the XP alleles D234N, S541R, Y542C, R601L, R683W, the XP/CS alleles G47R and G675R, and the TTD alleles R112H, R658C and R722W. These transgenes were able to rescue the null mutant and, because they were viable, they could be used for the various tests described below. Two suspected human null alleles, R616P and R616W, and, in addition, R683Q (a human XP allele), G602D (a human XP/CS allele), and R658H (a human TTD allele) did not rescue the fly null allele. The fact that both predicted human null mutations cannot rescue an *xpd*-null mutation in the fly system indicates that the system is capable of modeling the human *XPD* alleles. We have not ruled out that the lethality of the other lethal alleles is caused by an error, but it might also indicate that flies have a higher requirement for *xpd* activity than humans.

To determine the Xpd protein levels expressed from these transgenes we performed western blots on whole-fly extracts prepared from 1- to 3-day-old male flies and we normalized the Xpd protein levels with Bicaudal-D (BicD), an unrelated protein of similar mass (Fig. 1B,C). The two XP lines D234N and S541R and the XP/CS line G47R displayed slightly reduced Xpd levels compared to the wild-type line (62%, 58% and 69% of the *xpd^wt^* levels, respectively). Surprisingly, all three TTD alleles, R112H, R658C and R722W, gave slightly higher Xpd levels (128%, 151% and 145%, respectively). We were initially surprised by these results, because in human cells the TTD-type mutations in *XPD* cause a reduction of the steady-state levels of the p62, p44 and Cdk7 subunits of TFIIH (Botta et al., 2002; Botta et al., 2009) and it was assumed that the levels of the entire TFIIH levels were reduced, although Xpd levels were not assessed. As we will see later, the cTFIIH components that we assessed also showed reduced association with CAK in all *Drosophila* TTD mutants, indicating that the effect of the TTD mutations on (c)TFIIH stability is conserved between the systems. We conclude that, when expressed in *Drosophila*, the Xpd protein levels of the different mutants varied somewhat in 1- to 3-day-old males, but the level of variation was within a range that should allow us to detect differences in protein activities caused by the altered protein sequence.

### Lack of UV resistance in D234N (XP), G47R and G675R (XP/CS)

**Fig. 2.**
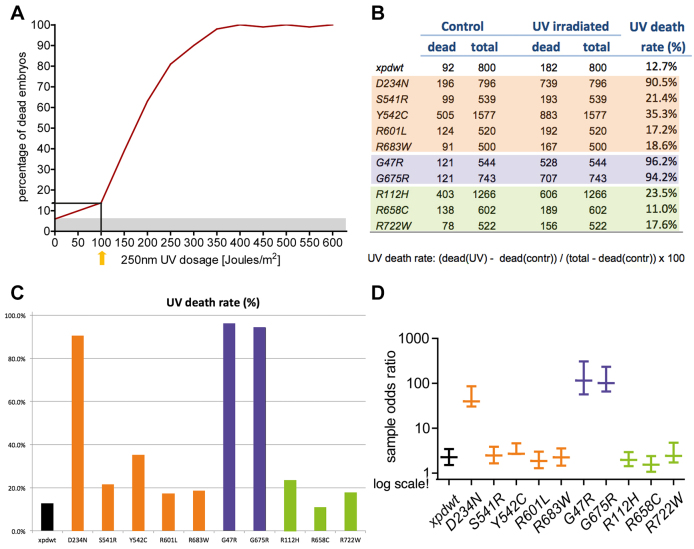
**UV sensitivity of *xpd* mutant embryos.** (A) UV sensitivity was measured by counting total and dead embryos. Embryos were UV-irradiated when they were 3–5 hours old. Wild-type embryos (*y w*) showed a death rate of 6% without irradiation and an increased death rate with the applied UV dose. Embryos did not survive more than 350 J/m^2^. (B) For the survival experiments of the different *xpd* alleles 100 J/m^2^ were applied. Half of the embryos were UV-irradiated when they were 3–5 hours old. The other half was not irradiated and served as a control. Homozygous embryos of the genotype *w; xpd^P^; xpd^test^, G147* were used to ensure that all embryos had the same maternal and zygotic genotype. Depicted are the numbers of dead embryos of each line and the total number of embryos for the control and for the irradiated group. (C) The UV-induced death rate, calculated from the data in B is also shown graphically. (D) The sample odds ratio of each *xpd* line with the corresponding simultaneous 95% confidence intervals are shown on a logarithmic scale. In comparison to the wild-type line, highly enhanced death rates and sample odds ratio were observed after UV irradiation for D234N (XP), G47R and G675R (both XP/CS).

To test the UV resistance of the different *xpd* alleles, 3- to 5-hour-old embryos of the different genotypes were UV-irradiated. As control, the same number of embryos of the same genotype was tested without irradiation. After 2 days, about twice the average time it takes to get through embryogenesis, dead embryos were counted in both groups. In order to find the optimal UV dose for the experiment, different UV dosages were tested with wild-type (*y w*) embryos. Without irradiation, 6% of the wild-type embryos died before they hatched as larvae (Fig. 2A). With increasing UV dosage, the death rate of irradiated embryos increased and no embryos survived 350 J/m^2^ or higher doses. For the survival experiments, we therefore chose 100 J/m^2^ as suitable test dose. *xpd^wt^* flies, bearing two copies of the wild-type *xpd* transgene, showed a mean death rate of 11.5% under normal development conditions without UV irradiation and one that was about twice this rate after UV irradiation with 100 J/m^2^ (22.75%; Fig. 2B,C). To assess the statistical significance of these results, the sample odds ratio of each *xpd* fly line was also determined (Fig. 2D). All but the R658C allele showed at least a slightly higher UV-induced lethality than the wild-type allele, and the strongest sensitivity towards UV irradiation was seen in the two XP/CS alleles G47R and G675R. Interestingly, human fibroblasts from XP/CS patients also show extremely high sensitivity to UV irradiation (25–50 times as sensitive as normal fibroblasts) (Taylor et al., 1997; Fujimoto et al., 2005). A possible interpretation of the data could be that the extremely high UV-induced death rate in the embryos with the XP/CS mutations is mainly caused by UV-induced strand displacement in XP/CS cells (Godon et al., 2012), whereas the modestly elevated rates seen in most XP and TTD mutants might reflect reduced NER. Interestingly, in cases where the human fibroblast lines allow an assessment of survival for a specific allele (R683W, R112H, R722W and possibly R601L, see below), similar UV sensitivities have been reported for XP and TTD mutants to those we see here (5×–12× elevated; Lehmann et al., 1988; Ichihashi et al., 1988; Broughton et al., 1994; Takayama et al., 1995; Takayama et al., 1996). The fibroblast line with the R601L allele carries, as the second allele, the D234N mutation. This XP line shows an eightfold elevated sensitivity, which is in the range of the XP alleles, and in our survival analysis, too, the R601L allele behaved like most other XP alleles. For the D234N allele, however, we found a sensitivity that was almost as high as the one of the XP/CS alleles. This might indicate that the R601L allele is dominant over D234N with regard to UV resistance. We also note that UV survival in *Drosophila* does not correlate with cancer prevention activity in humans, because the XP alleles that are clearly linked to tumor formation in humans, R683W and R601L (supplementary material Table S1), show fairly good survival after UV exposure of fly embryos.

### Altered TFIIH interactions caused by mutations in Xpd

**Fig. 3.**
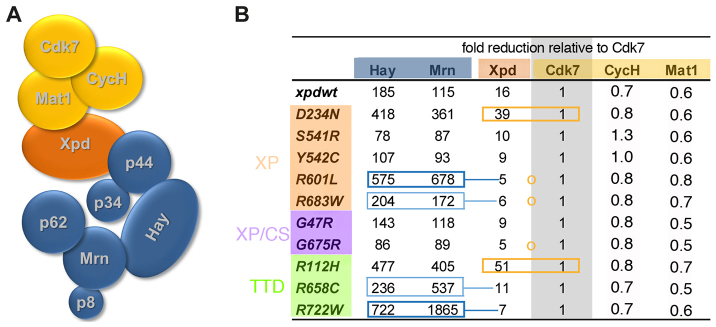
***xpd* mutations affect interactions in TFIIH.** (A) Model of TFIIH with its subunits. Upon isolation by anti Cdk7 antibody immunoprecipitations, Xpd and the cTFIIH subunits are found at lower concentration in the pellet. (B) The table shows the fold reduction by which Xpd, the two cTFIIH components Hay and Mrn, and the two additional CAK components are less abundant than Cdk7 in the anti-Cdk7 antibody immunoprecipitate from extracts of the mutants indicated. Data are derived from the detailed analysis shown in supplementary material Table S2. Framed in orange are the reduced interactions between Xpd and CAK. These mutants show a reduced relative detection frequency of Xpd and core components (compared to the wild-type *xpd* allele). Framed in blue are mutants that showed reduced detection frequencies of core components compared to Xpd, implying a reduced Xpd-cTFIIH binding. Dark blue indicates strong effects; light blue indicates weaker effects. Also indicated with orange circles are enhanced interactions between Xpd and CAK. Note that only one peptide each was analyzed for Hay and Mrn. However, because Hay and Mrn intensities react in similar ways to the different *xpd* mutant backgrounds, the intensities seem to reflect differences in levels and not modifications.

The large TFIIH complex, consisting of nine to ten polypeptides (Fig. 3A), is needed for full transcriptional activity (Egly and Coin, 2011). During NER, however, the CAK subcomplex is released from the TFIIH (Coin et al., 2008). On other occasions, the free trimeric CAK performs functions in the cell cycle, namely, the phosphorylation of the T-loop of cell cycle Cdks (Fisher, 2005). Different TFIIH functions therefore seem to be regulated in part through differences in complex composition, and Xpd plays an important physiological role in linking CAK to the cTFIIH (Chen et al., 2003; Chen and Suter, 2003). To test whether the different *xpd* mutations affect complex composition, anti-Cdk7 immunoprecipitations were performed on whole-fly extracts and TFIIH polypeptide levels were determined using targeted quantitative tandem mass spectrometry (qMS/MS) analysis. Table 1 lists the peptides that were chosen. Except for Hay (*Drosophila* Xpb) and Mrn (*Drosophila* p52), where only one peptide was used for the analysis, the sum of the intensities of the different peptides from three injections was calculated (supplementary material Table S2). All of them were standardized to the sum of the intensities of the Cdk7 peptides (Fig. 3B). Comparing the abundance of peptides from the CAK components (Cdk7, Cyclin H and Mat1) in the different *xpd* mutants revealed that this method is robust. The qMS/MS analysis revealed similar levels for these three polypeptides and, importantly, the abundance ratio between the three CAK components was constant throughout the different mutants. This result would be expected for anti-Cdk7 antibody immunoprecipitations if Cdk7 were primarily in complexes with Cyclin H and Mat1. Mutations in Xpd would not be expected to alter CAK stability, but some of the mutations might change the Xpd-CAK interaction, whereas others could reduce the interaction between Xpd and cTFIIH. A reduced Xpd:Cdk7 ratio, implying an impaired Xpd-CAK binding, was found in the mutants D234N (XP) and R112H (TTD; both framed in orange in Fig. 3B), whereas a higher ratio was seen in R601L, R683W (XP) and G675R mutants (XP/CS; Fig. 3B, orange circle between Cdk7 and Xpd), indicating that these mutations might stabilize the interaction. Impaired binding of Xpd to cTFIIH is suggested by the finding that core components were underrepresented in the immunoprecipitation relative to Xpd. This was observed in the two mutants R601L (XP) and R722W (TTD) and, to a lesser extent, also in R683W (XP) and R658C (TTD; all framed in blue). Remarkably, in all TTD alleles the levels of the cTFIIH component Mrn (and also Hay) were reduced compared to the Cdk7 levels, and that this reduction is at a level comparable with the reduced abundance observed in human TTD cells (Botta et al., 2002). This indicates that the TTD mutations destabilize cTFIIH binding to CAK or Xpd-CAK. It thus seems that a primary effect of TTD mutations in flies and humans could be the destabilization of TFIIH and, as a consequence, some TFIIH components show lower steady-state levels (e.g. human p52, p44, CDK7) whereas others do not (e.g. fly Xpd; see also Discussion).

**Table 1.**
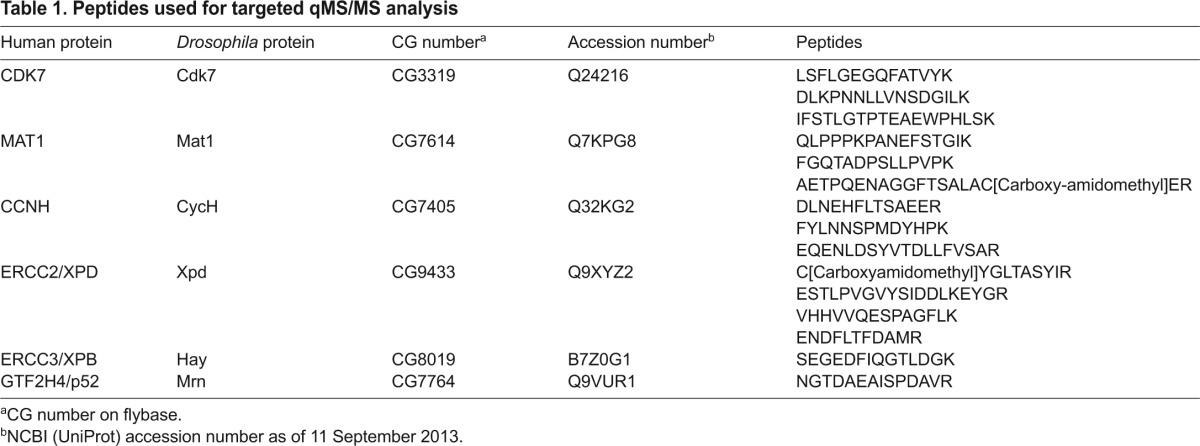
Peptides used for targeted qMS/MS analysis

### Interaction capacity of mutant human XPD with free human CAK

Xpd-mediated control of the cell cycle activity of CAK has been clearly demonstrated in young *Drosophila* embryos (Li et al., 2010). In the above section, we studied Xpd interactions in the presence of the entire TFIIH. However, the interaction between free XPD and CAK might be affected differently. We therefore also tested which *XPD* mutations specifically affected the interaction between mutant XPD and the CAK subcomplex. Such a weaker interaction had been described for the R683W XP allele (Keriel et al., 2002) and a study using a yeast two-hybrid system has shown that the TTD mutant R112H retained 90%, the XP mutant D234N 55% and the XP/CS mutant G602D 60% of the interaction capacity (Sandrock and Egly, 2001). We therefore used this yeast system to test specifically the interaction capacity of the mutant forms of human XPD with the human CAK complex as described previously (Sandrock and Egly, 2001). In this assay, we could also test the interaction capacity of the mutants that did not support viability in flies and we also included one null allele (R616P). The results of this experiment are shown in supplementary material Fig. S1. XPD binds to the Mat1 subunit of CAK through its N-terminal domain, and to p44 of the cTFIIH through its C-terminal domain (Fig. 1A; Schultz et al., 2000; Sandrock and Egly, 2001). Surprisingly, the interaction with CAK is not only affected by a mutation in the HD1 domain in the N-terminal half of XPD (where Mat1 binds), but also by mutations in the C-terminal half, in particular in the center of HD2 or towards the groove. Interestingly, whereas two clearly carcinogenic mutations, R683W and R601L, showed a clear reduction in the XPD-CAK interaction (down to about 44% and 33%, respectively, of the normal interaction capacity), the third one, R683Q, displayed an elevated interaction capacity with CAK.

Aside from the hypomorphic mutant forms of human XPD, we also tested one null mutant and two frequently occurring polymorphisms, D312N and K751Q, that have been studied intensively for their possible association with cancer susceptibility (reviewed in Clarkson and Wood, 2005; Manuguerra et al., 2006). Interestingly, whereas the ‘null’ mutant bound strongly to CAK, the two *XPD* polymorphisms also showed a clear reduction in their interaction capacity with CAK (supplementary material Fig. S1). D312N retained only about 51% of the normal interaction level, whereas K751Q showed ~60%.

### *In vivo* cell cycle effects of *xpd* mutants

**Fig. 4.**
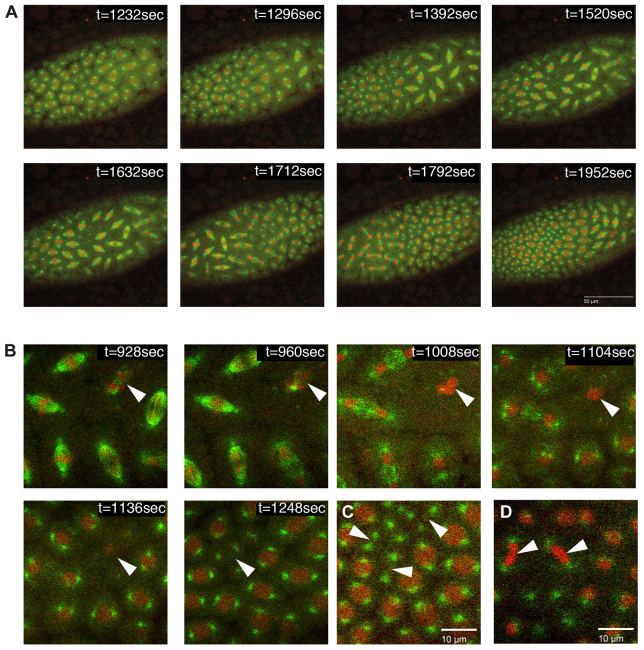
**Cell cycle defects scored in *xpd* mutants.** *G147*-GFP (green) marks the mitotic spindle while Histone-RFP (red) labels the chromatin. Genotype of the embryos was *w; xpd^P^/ Df (2R)PF1; P{His2Av-mRFP1}II.2; xpd^test^, G147/ +*. Note that picture acquisition was optimized for live imaging to reduce phototoxicity. (A) Asynchronous cell cycle in G47R (XP/CS) during cycle 11. Cell cycle phases progressed unidirectionally instead of bidirectionally from both poles. In this case, the mitotic wave started at the posterior end on the right side. (B) Arrowheads point to a sinking nucleus in cell cycle 10 in a G47R (XP/CS) embryo. The spindle was not built up properly and, in the following interphase, the nucleus sunk into the interior of the embryo, eliminating the DNA from the cortex. The two centrosomes from this mitotic figure stayed at the surface. The numbers indicate seconds into the movie. (C) Arrowheads point to free centrosome pairs at the surface of the embryo, most likely caused by extrusion of the nuclei in a previous cycle. Often the centrosomes keep dividing in subsequent cell cycles. The embryo carried a G47R (XP/CS) mutation and was in interphase between cycle 11 and 12. (D) R683W (XP) nuclei failed to divide and the chromatin stayed together after anaphase of cycle 12. Persisting chromatin bridges are pointed out by arrowheads.

**Fig. 5.**
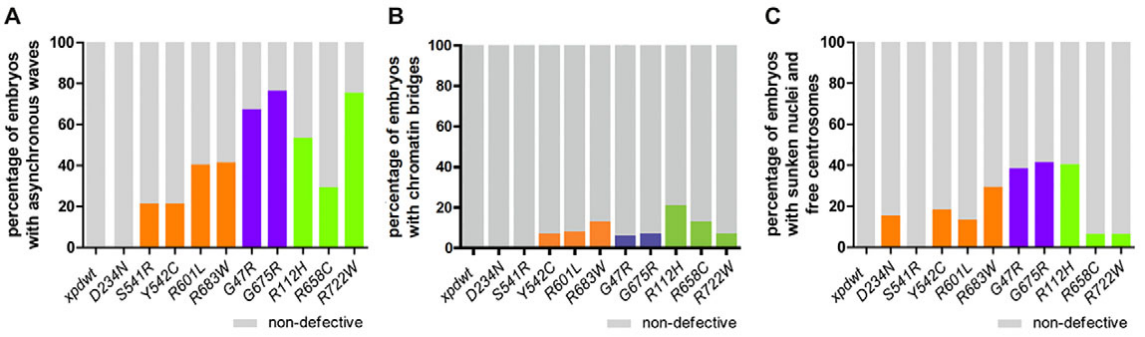
**Frequency of cell cycle defects in the *xpd* mutants.** Percentage of embryos showing cell cycle defects in the different *xpd* mutant embryos. Only the first defect observed was scored in each nucleus. (A) Percentage of embryos with asynchronous mitotic waves, indicating lack of coordination of the nuclear divisions. (B) Frequency of embryos that show first a chromosome segregation defect (chromatin bridges). (C) Loss of nuclei and chromosomes was scored by counting sunken nuclei and free centrosomes.

It has been demonstrated that Xpd has transcription independent and essential functions in cell cycle regulation in *Drosophila* during the embryonic cleavage divisions (Li et al., 2010). In this process, Xpd regulates proper distribution and activity of CAK, and embryos lacking Xpd show a series of nuclear division defects. We therefore set out to investigate whether some *xpd* mutations caused similar cell cycle phenotypes as seen in embryos lacking Xpd. Several different defects were scored for the different *xpd* mutant alleles, but only defects that arose in a nucleus that previously did not show defects were counted for the statistics (Figs 4, 5). Using a live-imaging approach allowed us to apply this condition and to focus on the primary defects. Table 2 summarizes the number of embryos that were analyzed and used for each genotype.

**Table 2.**
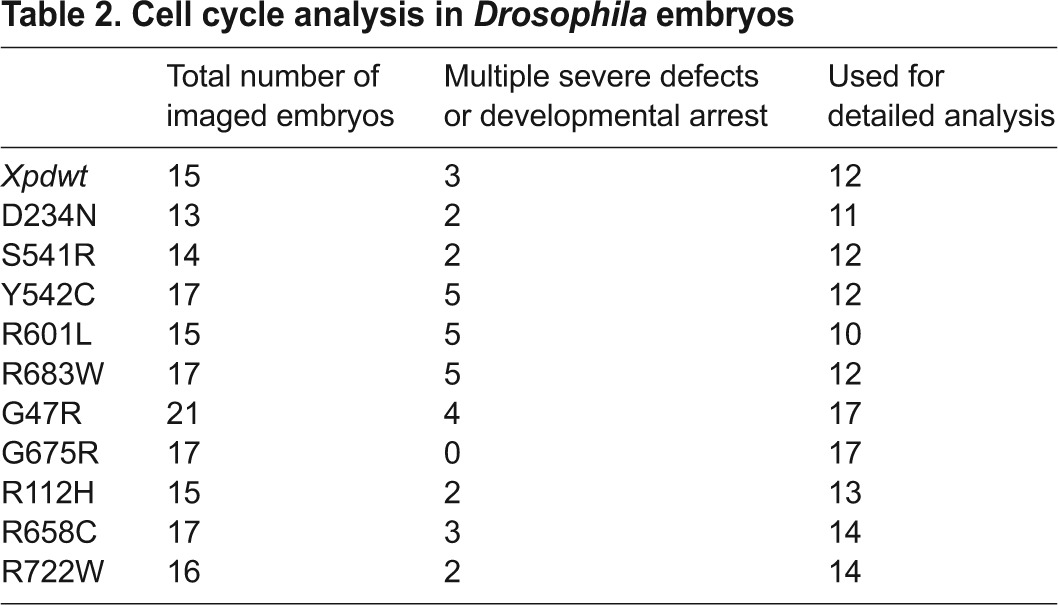
Cell cycle analysis in *Drosophila* embryos

Wild-type embryos divide metachronously during the later syncytial mitoses. Starting during nuclear cycle 10, the almost synchronous divisions can be resolved into two rapid mitotic waves, originating bidirectionally from both embryonic poles and proceeding towards the center (Foe and Alberts, 1983). This process presents a fascinating model that could reveal how cells can use environmental cues to control cell cycle progression. So far, however, very little is known about this synchronization control, but in embryos lacking Xpd, mitotic synchronization is disrupted and a different type of wave is detected. Nuclear divisions start at only one pole and almost reach the second pole before the divisions start there too (Li et al., 2010; also see Fig. 4A). Analysis of the synchrony of early mitotic cycles revealed that the wild-type *xpd^wt^* transgene was able to rescue this phenotype and *xpd^wt^* embryos divided synchronously and metachronously (Fig. 5A).

Several of the mutant alleles tested were unable to rescue the synchronization defect. Accompanying the loss of mitotic synchrony, cleavage divisions slowed down considerably, and we observed mitotic waves starting from either pole and almost reaching the other pole before the mitotic waves were initiated there. Clearly elevated synchrony defects were detected in the following mutant embryos: R601L and R683W (both XP), the XP/CS mutants G47R (>60%) and G675R (>70%), and in the TTD alleles R112H (50%) and R722W (>70%; Fig. 5A). The capability of Xpd to synchronize thousands of nuclear divisions in a timely fashion was severely hampered in particular by four mutations, including both XP/CS alleles studied. The correlation between the loss of cell cycle coordination and neuro-developmental phenotypes in human patients will be discussed below.

Live-imaging analysis of the mitotic divisions of the embryonic nuclei showed how well the different mutant forms of Xpd supported the different cell cycle control functions during the nuclear division cycles. Several mutants caused the formation of chromatin bridges between mitotic nuclei (Fig. 5B), as seen when embryos develop without detectable Xpd (Li et al., 2010). Severe problems in chromosome segregation (and possibly other problems) can cause loss of nuclei from the surface of the embryo and simultaneously lead to areas with free centrosomes that often continue to divide in subsequent cycles (Li et al., 2010; Sullivan et al., 1990; Sullivan et al., 1993). Because of the difficulty of detecting, in particular, small chromatin segregation problems, we also list the frequency of embryos where the first phenotype was either embryos with sunken nuclei or areas of free centrosomes (Fig. 5C). Loss of nuclei is occasionally also seen in wild-type embryos (although at lower frequency and not in this experiment). An enhanced rate of DNA loss and free centrosomes was detected for the XP mutant R683W (29%), for both XP/CS mutants, G47R (38%) and G675R (41%), and for the TTD mutant R112H (40%). Of these mutants, R683W is clearly linked to high cancer risk and G47R is possibly linked too, but R112H is much less linked to cancer risk (Takayama et al., 1995; Taylor et al., 1997; Botta et al., 2002; Ueda et al., 2009).

## DISCUSSION

### The Drosophila Xpd model

Our *Drosophila* model allows us to test the effect of any human *XPD* mutation on the various cellular functions of Xpd. Two control mutants that are described as null alleles in humans, R616P and R616W, did not rescue the lethal *xpd*-null phenotype in *Drosophila*, whereas most of the other mutant *xpd* alleles did. This shows that the human *XPD* and the *Drosophila xpd* mutations behave similarly, and the fly system therefore seems to be an adequate model. Inserting the transgenic copies of mutant *xpd* into the same site of an attP landing platform allowed us to obtain similar protein expression levels from all the different transgenes (Fig. 1B,C), an important requirement for comparing the effects of individual amino acid substitutions on gene and protein function. We therefore assume that the observed effects of the mutations reflect alterations in protein functional activity and not levels. This provides a good basis to assess the effect of an amino acid alteration on the performance of the protein in its different cellular activities. We found reduced levels of core TFIIH components in Cdk7 immunoprecipitations from all three TTD mutants tested, and in one of them, R112H, Xpd levels were also reduced (Fig. 3B). Studies in cells from human patients have shown that TTD type mutations in *p8*, *XPB* and *XPD* caused markedly reduced levels of TFIIH components p62, p44 and Cdk7, suggesting that TTD mutations destabilize the TFIIH complex in human cells (Botta et al., 2002; Botta et al., 2009; Vermeulen et al., 2000). In total extracts from the TTD mutant flies, Xpd levels were not reduced (and were even slightly higher than in the wild type), but there was a clear effect of all TTD mutations on *Drosophila* TFIIH stability (Fig. 3). It therefore seems that the primary effect of TTD mutations is on TFIIH stability and that this effect is conserved between human and flies. The reduced TFIIH stability then leads, for some subunits, to a reduced steady state level (e.g. human p62, p44, Cdk7), whereas for others, these levels seem not to be reduced (e.g. *Drosophila* Xpd). The effect on the steady-state levels might also be influenced by different culture temperatures (flies were kept at 25°C, whereas human cells were grown at 37°C) or by the different behavior of the mutant protein, which could affect its biochemical isolation. Interestingly, the R658C (TTD) mutation is indeed known to be temperature sensitive, and human TFIIH complex stability is sensitive to elevated temperature (Vermeulen et al., 2001).

Human XP-D patients show severe phenotypes, and mutations in the *Drosophila* genes *hay* and *mrn*, encoding the cTFIIH component Xpb and p52, respectively, have many visible phenotypes, and even under normal, unchallenged growth conditions, mutations cause higher apoptotic levels that lead to the development of defective wings (Merino et al., 2002; Fregoso et al., 2007). Like *hay* and *mrn*, *xpd* is also an essential gene in flies (Mounkes et al., 1992; Fregoso et al., 2007; Li et al., 2010) and deficiency for XPD is also lethal in mice (de Boer et al., 1998). The lethality in XPD-deprived animals might be due to their failure to start zygotic transcription (de Boer et al., 1998; Li et al., 2010). Surprisingly, however, young flies expressing only a hypomorphic Xpd that mimics a human mutant form do not show visible deformations of wings, eyes or bristles, and these young flies also move normally (data not shown). The differences between mutations altering the Xpd helicase or the Xpb helicase might be due to the fact that the ATPase or helicase activity of Xpb is essential for transcription, whereas the helicase activity of Xpd is not (Winkler et al., 2000). Indeed, mutations that reduce the helicase activity of Xpb affect bristles and body shape (Merino et al., 2002). From this, we suggest that most of the mutations that we examined in fly Xpd provide enough structural support to function in TFIIH-dependent transcription, an aspect that we did not study directly in this work. In addition, it seems that, under the protected laboratory environment, the basal level NER function in these *xpd* mutants is sufficient to prevent induction of high levels of apoptosis during development. It will be interesting to find out whether this changes when the flies are challenged with mutagens. We also performed initial experiments to find out whether any of these alleles show an advanced aging phenotype. Indeed, experiments designed to test the flight and walking capabilities seem to indicate that at least flies expressing the TTD allele R112H aged prematurely.

### Synchrony problems in early embryonic cell cycles correlate with neurological and developmental abnormalities in human XP-D patients

The cell cycle coordination function of *xpd* is strongly affected by XP/CS and TTD mutations (Fig. 5A). It is common for XP/CS and TTD patients to display neurological abnormalities and developmental defects (Kraemer et al., 2007; Lehmann et al., 2011), suggesting a possible link between these phenotypes and the regulation of cell division timing mediated by Xpd. The two XP/CS alleles G47R and G675R, as well as the TTD allele R722W, showed the highest frequency of asynchronous waves of all the *xpd* mutants in our *Drosophila* model (Fig. 5A). Human patients with these mutations display severe neurological abnormalities, reduced growth, and delayed and defective development (supplementary material Table S1; Bergmann and Egly, 2001; Botta et al., 2002; Broughton et al., 1995; Viprakasit et al., 2001; Theron et al., 2005; Fujimoto et al., 2005). Strikingly, the frequency of detected asynchronous waves correlates very well with the degree of neurological and developmental abnormalities in human TTD patients. Compared with other TTD patients, patients with the R658C mutation show only mild neurological and developmental defects (Bergmann and Egly, 2001), and R658C flies showed far fewer asynchronous waves than the other TTD alleles. Among the XP-type patients, those with an R683W mutation show the most severe neurological abnormalities (Ichihashi et al., 1988; Taylor et al., 1997) and, in flies, R683W also gave the highest frequency of asynchronous waves, together with R601L.

The XP allele D234N did not display any mitotic asynchrony. However, because this allele displayed strongly disrupted binding of Xpd to the CAK complex in one experiment (Fig. 3) and reduced binding in another experiment (supplementary material Fig. S1), we further conclude that the role of Xpd in synchronization of the cell cycle for the thousands of nuclei does not strongly depend on proper Xpd-CAK interaction. This is a significant and unexpected finding, because the other cell cycle function of Xpd is to titrate CAK and its activity at the right time in the cell cycle (Chen et al., 2003; Li et al., 2010).

### Altered XPD interactions in TFIIH

XPD acts as a bridge between the cTFIIH and CAK subcomplexes, and its physical interactions with other TFIIH subunits therefore affect its different functions (Cameroni et al., 2010). Cdk7 is present in the free trimeric CAK complex with Mat1 and Cyclin H, in the tetrameric CAK complex with Xpd as the forth subunit, and also in holo-TFIIH. The qMS/MS approach allowed us to estimate the relative interaction between different Xpd variants and their TFIIH partners by measuring the relative abundance of the subunits of the Cdk7 complexes. Among the different *xpd* mutants we found over-representation of the trimeric CAK complex for the D234N (XP) and R112H mutations (TTD; Fig. 3B), pointing to impaired Xpd-CAK binding in these mutants. Both mutations alter residues that map to the interaction domain between Xpd and the CAK subunit Mat1 (Fig. 1; Fan et al., 2008; Schultz et al., 2000; Sandrock and Egly, 2001). Surprisingly, the mutations strongly linked to tumor formation, XP alleles R601L and R683W, altered the interactions of Xpd with both the CAK and cTFIIH subcomplexes (Fig. 3B). Our results showed an over-representation of the tetrameric CAK complex among the different Cdk7 complexes (indicative of enhanced interactions between Xpd and CAK). In addition, these two mutants also showed a strong and a medium reduction, respectively, of the interaction between the mutant Xpd and cTFIIH.

In contrast to the fly result, the yeast two-hybrid test revealed that the two XP alleles that are linked to high cancer risk reduced the interaction capacity between XPD and CAK (supplementary material Fig. S1). The differences between the fly and the yeast results might be due to the different origins of the proteins (fly genes and proteins were tested in the fly, but human genes and proteins in the yeast system) or to different experimental conditions (e.g. temperature, salt, extraction). An interesting difference is also that, in the yeast experiment, human Xpd is expected to primarily associate with the trimeric human CAK complex, whereas, in the *Drosophila* experiment, cTFIIH and possibly MMS19 and MIP18 also compete for Xpd. A reduced Xpd-cTFIIH interaction might then also contribute to an increased binding of Xpd to CAK in the fly.

In our *Drosophila* assay, R722W (TTD) and R601L (XP) and, to a lesser extent, R683W (XP) and R658C (TTD) reduced the interaction of Xpd with cTFIIH. For three of these mutations, R722W, R683W and R658C, a different approach also showed reduced XPD-p44 interaction (Dubaele et al., 2003) and for R658C, patient information shows that the mutation causes a weakening of the TFIIH interaction under certain conditions. The R658C (TTD) mutation is temperature sensitive in humans, causing patients to lose hair when they contract a fever (Vermeulen et al., 2001). Indeed, transcription, the repair function and TFIIH complex stability are more sensitive to elevated temperature in R658C patients than in other TTD patients. Interestingly, among the mutants that show high frequencies of chromosomal loss and free centrosomes, the ones that also show strongly reduced Xpd-cTFIIH interaction and also altered Xpd-CAK interaction (R601L, R683W) are the ones that are particularly strongly associated with high cancer risk.

The fly Xpd model presented here is capable of modeling different aspects of the very complex syndromes associated with mutations found in human patients. Using this model, we found an interesting positive correlation between problems in control of the synchrony of the early embryonic nuclear divisions and neurological and developmental abnormalities in human XP-D patients. Furthermore, we propose that Xpd-TFIIH and CAK interactions should be considered as risk factors for cancer development, in particular in cases where the mutation leads to high rates of chromatin loss and free centrosomes. An interesting, but demanding, extension of this project will be to produce flies that express the same allelic combinations found in human XP-D patients. With these, researchers will be able to test the effect of any combination of two different alleles on the various *xpd* phenotypes.

## MATERIALS AND METHODS

### DNA cloning and constructs

Mutant human *XPD* cDNA constructs were derived from pXPD-VP16 (Sandrock and Egly, 2001). The fly genomic *xpd* DNA, cloned into pSL1180 and pCaSpeR (Li et al., 2010), was used to produce the different fly *xpd* alleles through PCR mutagenesis (Sambrook and Russel, 2001). All sequences generated by PCR were verified by sequencing.

### Fly stocks

Transgenic stocks were established with the *attP*-landing platform *64A* and *ΦC31*-based integration (Bischof et al., 2007). For UV survival experiments and TFIIH stability assays, embryos and flies, respectively, were homozygous for the *xpd^P^* allele and for the transgenic *xpd* copies. For the cell cycle analysis, we used embryos from mothers bearing the *xpd^P^* null allele over Df(2R)PF1, which lacks the *xpd* gene (Li et al., 2010). The hemizygous configuration avoided making second site hits on this chromosome homozygous. The deficiency chromosome was recombined with histone *His2Av-mRFP* (Bloomington).

### UV sensitivity assay

The day before collecting eggs, flies were transferred into small cages and placed onto apple juice plates containing a drop of fresh yeast. For the collection, apple juice plates were replaced with fresh ones and flies were allowed to lay eggs for 2 hours. Plates were then removed and incubated for another 3 hours at 25°C. Groups of 100 3- to 5-hour-old embryos were used either as controls or irradiated in the UV Stratalinker^®^ 2400 with a 254 nm light source at 100 J/m^2^. Embryos were kept in the dark, transferred onto fresh fly food and incubated at 25°C for 48 hours. Dead embryos were then counted. For the statistical analysis the sample odds ratio of the non-irradiated control and the irradiated sample was calculated for each fly line.

### Immunoprecipitations, qMS/MS and western blots

Four 2 ml Eppendorf tubes were filled with 1- to 3-day-old flies and frozen in liquid nitrogen. Subsequent storage was at −80°C. Frozen flies were homogenized with a pestle in a pre-cooled porcelain bowl containing liquid nitrogen. The fly powder was then transferred into a 15 ml falcon tube and 3 ml lysis buffer (50 mM Hepes pH 7.4, 150 mM NaCl, 1 mM EDTA pH 8.0, 0.1% Triton X-100, 50 mM NaF, 80 mM sodium β-glycerophosphate, 1 mM PMSF and EDTA-free protease inhibitor cocktail; Roche) were added. Extraction was with a glass homogenizer, and extracts were incubated for 30 minutes on ice. Samples were then centrifuged at 4°C and 16,000 ***g*** for 10 minutes and the aqueous phase was transferred into 1.5 ml Eppendorf tubes and centrifuged again in the same way. Bradford assays were subsequently performed with the pooled clear extracts. In the meantime, proteins were stored in 15% glycerol at −20°C.

For each immunoprecipitation, 80 μl GammaBind™ Plus Sepharose™ beads (GE Healthcare) were incubated on a slowly rotating wheel for 2 hours at room temperature with 120 μg mouse anti-Cdk7 (20H5) and 120 μg mouse anti-Cdk7 (19E7; both Larochelle et al., 1998). For control sample 1, the 80 μl Sepharose beads were only washed with lysis buffer; for control sample 2, the beads were incubated with 1.5 ml mouse anti-BicD (1B11; Suter and Steward, 1991). 25 mg total fly extracts were then used for each immunoprecipitation. Extracts were added to 80 μl pre-treated Sepharose beads and incubated for 2 hours at 4°C while slowly rotating. Beads were washed twice with 500 μl wash buffer 1 (50 mM Hepes, 150 mM NaCl, 1 mM EDTA, 50 mM NaF, 80 mM sodium β-glycerophosphate, 1 mM PMSF and protease inhibitor cocktail, pH 7.4; Roche). 1 ml wash buffer 1 was added and beads were incubated for 10 minutes at 4°C on a slowly rotating wheel and subsequently washed once with wash buffer 2 (50 mM Hepes, 100 mM NaCl, 1 mM EDTA, 50 mM NaF, 80 mM sodium β-glycerophosphate, 1 mM PMSF and protease inhibitor cocktail, pH 7.4). The buffer was completely removed and 20 μl NuPAGE^®^ LDS sample buffer (Novex^®^, Invitrogen) was added. Samples were boiled and loaded on an 8% SDS PAGE gel. Electrophoresis was stopped when samples had run ~1 cm into the resolving gel. The gel was washed with milli-Q water and Coomassie staining was performed using the Novex^®^ Colloidal Blue Staining Kit (Invitrogen).

The uppermost band of the gel was then removed and each remaining sample was divided into 5 slices. Each slice was cut into 4–6 cubes of 1 mm^3^, transferred into a 1.5 ml Eppendorf tube, covered with 100 μl 20% ethanol and stored at 4°C. Peptide ions with defined charge state were selected from a list of representative peptides for the different TFIIH complex proteins found in the *Drosophila* peptide atlas (Table 1). These peptides were isolated using a time-scheduled inclusion mass list (Loevenich et al., 2009). Proteins were reduced, alkylated and digested by trypsin as described previously (Gunasekera et al., 2012). The digests were analyzed by liquid chromatography (LC)-MS/MS (EASY-nLC 1000 coupled to a QExactive mass spectrometer, ThermoFisher Scientific) with three repetitive injections of 5 μl digests. Peptides were trapped on an Acclaim PepMap100 C18 pre-column (3 μm, 100 Å, 75 μm×2 cm, ThermoFisher Scientific, Reinach, Switzerland) and separated by backflush on a C18 column (5 μm, 100 Å, 75 μm×7.5 cm, Magic C18 HomeMade) by applying a 60-minute gradient of 5% acetonitrile to 40% in water, 0.1% formic acid, at a flow rate of 400 nl/min. Targeted peptides were detected on the QExactive with resolution set at 70,000 with an automatic gain control (AGC) target of 5E05 and maximum ion injection time of 250 ms. The data-dependent method for precursor ion fragmentation was applied with the following settings: resolution 17,500, AGC of 2E05, maximum ion time of 120 milliseconds, mass window 2 *m*/*z*, underfill ratio 0.1%, charge exclusion of unassigned and 1+ ions, and peptide match on, respectively. Peptide quantification values were accepted when the CV value, which is the coefficient of variation of three injections, was below 50%. Pinpoint software (Thermo Scientific, version 1.2) was used. The identical set of transitions was used across all samples to define protein quantities.

For western blot analyses, affinity purified rabbit anti-Xpd antibody (Chen et al., 2003) was used at 1:500 overnight at 4°C. Secondary antibodies were IRDye 680CW-conjugated goat anti-rabbit 1:15,000 and IRDye 800CW-conjugated goat anti-mouse 1:15,000 (both from LI-COR).

### Yeast four-hybrid experiments

Yeast four-hybrid experiments were performed as described in detail previously (Sandrock and Egly, 2001). Briefly, human Cdk7 was fused to the DNA-binding domain Lex9 and human XPD to the activation domain VP16. Human Cyclin H and MAT1 were also expressed to allow the formation of the stable ternary CAK complex. If the stable complex forms, LexA-Cdk7 binds to the DNA-binding element of the reporter genes *HIS3* and *lacZ* and the VP19 domain recruits the transcription factors. The interaction strength can be determined by analyzing the resulting β-galactosidase activity. Mutant human *xpd* cDNA constructs were derived from pXPD-VP16 (Sandrock and Egly, 2001). All sequences generated by PCR were verified by sequencing.

### Cell cycle analysis

Embryos were collected as described above, but only for 45 minutes. They were then immediately washed and dechorionated with diluted bleach, and washed again with tap water. Four rows of 12 embryos were then lined up and transferred onto a coverslip with a stripe of glue [one role of double side sticking tape (Migros) dissolved in 50 ml heptane]. Embryos were then covered with Voltalef oil 10S (VWR International). Imaging was done with the confocal laser scanning microscope TCS SP5 (Leica Microsystems) using the resonant scanner in bidirectional mode, the 63× objective and a HyD. During the live-imaging the stage temperature was set at a constant 25°C. GFP and RFP signals were excited with the 488 and 561 nm lasers. Four embryos were imaged every 16 seconds using a line-average of five and a frame-average of one. Imaging of each embryo consisted of eight sections with a *z*-distance of 10–12 μm, and each section was an image of 1024×1024 pixels. Images were further processed by applying a maximum projection and exporting the picture series as movie files. For analysis the movie files were imported into Adobe Photoshop CS6.

## Supplementary Material

Supplementary Material
