# Supplementary material for: A Drosophila XPD model links cell cycle coordination with neuro-development and suggests links to cancer
Source: Dis Model Mech. 2014 Nov 27;8(1):81–91. doi: 10.1242/dmm.016907 (PMC4283652; doi:10.1242/dmm.016907)
Supplement: Supplementary Material [file supp_8_1_81__index.html]

A Drosophila XPD model links cell cycle coordination with neuro-development and suggests links to cancer — Supplementary Material 

# A *Drosophila* XPD model links cell cycle coordination with neuro-development and suggests links to cancer

## DMM016907 Supplementary Material

**Files in this Data Supplement:**

- **Supplementary Material**
