# Supplementary material for: A Drosophila XPD model links cell cycle coordination with neuro-development and suggests links to cancer
Source: Dis Model Mech. 2014 Nov 27;8(1):81–91. doi: 10.1242/dmm.016907 (PMC4283652; doi:10.1242/dmm.016907)
Supplement: Supplementary Material [file supp_8.1.81_DMM016907.pdf]

Supplementary material Fig S1:

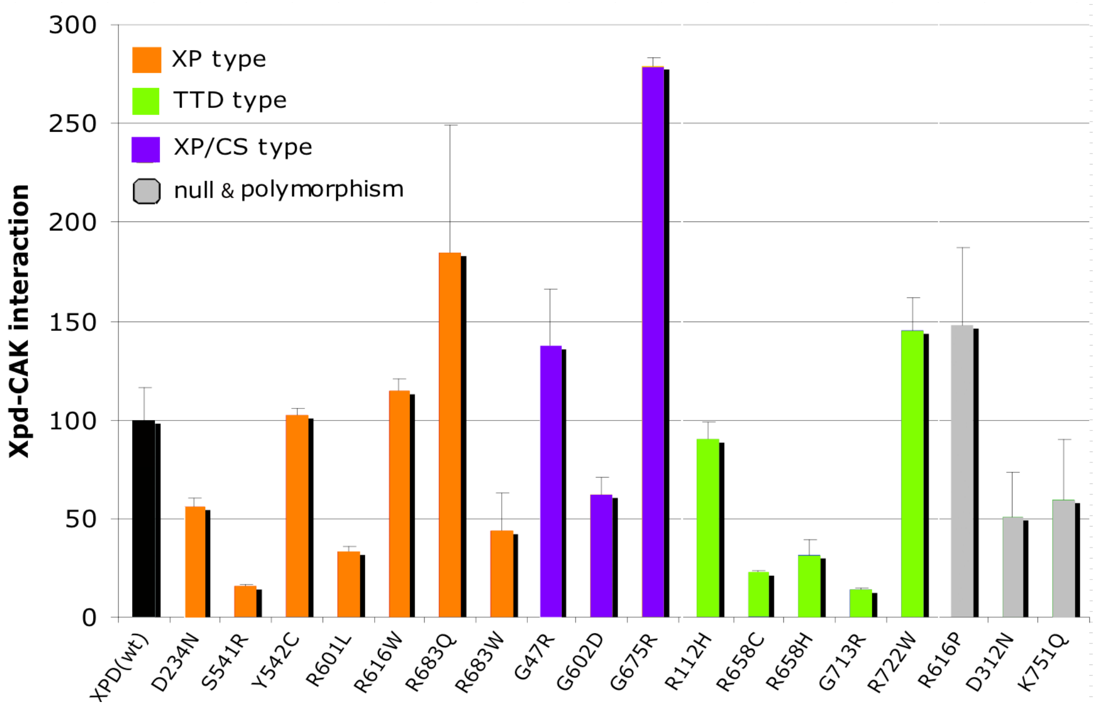

Yeast 2+2 hybrid interactions between human XPD variants and human CAK

Different mutant human XPD proteins showed variable XPD-CAK interaction capacity. Values for XPD-CAK interactions were normalized to the one of the wild type XPD (100%). Experiments for wild type XPD were repeated 10 times, for *R683W* (XP) and the two polymorphisms 7 times, for *D234N* (XP), *G47R* and *G602D* (XP/CS) and *R112H* (TTD) 4 times, and for the remaining ones 2 times.

Two carcinogenic mutations, *R683W* and *R601L*, showed a clear reduction in XPD-CAK interaction (down to about 44% and 33%, respectively, of the normal interaction capacity), while the third one, *R683Q,* displayed an elevated interaction capacity with CAK. The XP mutation *S541R* caused the strongest reduction, but there is insufficient patient data for this mutation (Supplementary Material Table S1; Kobayashi et al., 1997). *D234N* also showed a reduction, while *Y542C* and *R616W* displayed a normal interaction capacity with CAK.

The XP/CS allele *G602D* also showed a reduction in XPD-CAK interaction and the single patient with this allele contracted several tumors (Supplementary Material Table S1). The *G675R* mutant, on the other hand, displayed a very strong CAK interaction, but for this mutation there is not sufficient patient data (Broughton et al., 1995; Theron et al., 2005). The two most frequent TTD mutants, *R112H* and *R722W,* displayed almost normal or increased XPD-CAK interactions.

Broughton, B. C., Thompson, A. F., Harcourt, S. A., Vermeulen, W., Hoeijmakers, J. H., Botta, E., Stefanini, M., King, M. D., Weber, C. A., Cole, J. et al. (1995) 'Molecular and cellular analysis of the DNA repair defect in a patient in xeroderma pigmentosum complementation group D who has the clinical features of xeroderma pigmentosum and Cockayne syndrome', Am J Hum Genet 56(1): 167-74.

Kobayashi, T., Kuraoka, I., Saijo, M., Nakatsu, Y., Tanaka, A., Someda, Y., Fukuro, S. and Tanaka, K. (1997). Mutations in the XPD gene leading to xeroderma pigmentosum symptoms. Hum. Mutat. 9, 322-331.

Theron, T., Fousteri, M. I., Volker, M., Harries, L. W., Botta, E., Stefanini, M., Fujimoto, M., Andressoo, J. O., Mitchell, J., Jaspers, N. G. et al. (2005) 'Transcription-associated breaks in xeroderma pigmentosum group D cells from patients with combined features of xeroderma pigmentosum and Cockayne syndrome', Mol Cell Biol 25(18): 8368-78.

Table S1.

Download Table S1

Table S2.

Group value of total area of three injections and corresponding CV value in %

Protein	Accession number[a]	Peptide	Retention time	*xpdwt*		*D234N*		*S541R*		*Y542C*		*R601L*		*R683W*		*G47R*		*G675R*		*R112H*		*R658C*		*R722W*		anti-BicD IP		IP without antibody	
				Total area	CV	Total area	CV	Total area	CV	Total area	CV	Total area	CV	Total area	CV	Total area	CV	Total area	CV	Total area	CV	Total area	CV	Total area	CV	Total area	CV	Total area	CV
Cdk7	Q24216	LSFLGEGQFATVYK	42.96	3.06E+07	4	2.20E+07	3	2.44E+07	3	1.43E+07	6	4.58E+07	2	2.53E+07	2	1.56E+07	1	1.40E+07	11	2.39E+07	19	1.88E+07	5	2.03E+07	2				
		DLKPNNLLVNSDGILK	39.78	1.11E+07	3	6.55E+06	3	8.42E+06	9	5.53E+06	3	2.92E+07	3	1.47E+07	3	7.16E+06	4	7.15E+06	17	9.63E+06	41	7.63E+06	4	8.59E+06	8				
		IFSTLGTPTEAEWPHLSK	40.54	1.07E+07	4	6.36E+06	6	6.90E+06	12	3.22E+06	30	2.38E+07	1	1.29E+07	4	6.05E+06	7	5.28E+06	33	9.75E+06	32	7.74E+06	2	6.81E+06	3				
Mat1	Q7KPG8	QLPPPKPANEFSTGIK	28.14	2.25E+07	2	7.88E+06	1	5.08E+06	34	3.07E+06	5	2.82E+07	5	1.35E+07	9	7.75E+06	14	8.52E+06	12	1.12E+07	29	1.04E+07	31	1.04E+07	2				
		FGQTADPSLLPVPK	37.9	5.11E+07	2	4.13E+07	0	4.59E+07	2	2.70E+07	6	7.95E+07	2	4.62E+07	4	3.88E+07	5	3.66E+07	16	3.74E+07	23	4.36E+07	27	4.02E+07	0				
		AETPQENAGGFTSALAC[Carboxyamidomethyl]ER	32.21	1.68E+07	2	1.40E+07	5	1.09E+07	38	9.90E+06	9	2.43E+07	2	1.48E+07	5	1.03E+07	2	9.83E+06	12	1.13E+07	19	1.25E+07	20	1.37E+07	3				
CycH	Q32KG2	DLNEHFLTSAEER	33.55	3.78E+07	9	2.12E+07	2	1.31E+07	43	1.05E+07	1	6.16E+07	10	3.45E+07	13	1.90E+07	3	1.62E+07	13	2.62E+07	32	3.04E+07	29	2.69E+07	3			8.19E+04	16
		FYLNNSPMDYHPK	31.33	1.06E+07	5	5.88E+06	7	3.49E+06	46	3.69E+06	1	2.23E+07	1	1.13E+07	4	4.92E+06	2	5.37E+06	15	8.03E+06	27	6.39E+06	24	6.30E+06	2				
		EQENLDSYVTDLLFVSAR	53.39	2.25E+07	2	1.33E+07	7	1.45E+07	3	8.78E+06	3	4.03E+07	2	1.94E+07	5	1.07E+07	9	1.19E+07	12	1.80E+07	10	1.18E+07	11	1.50E+07	2			6.01E+04	62
Xpd	Q9XYZ2	C[Carboxyamidomethyl]YGLTASYIR	32.43	7.14E+05	4	4.05E+05	5	1.50E+06	30	9.44E+05	7	4.55E+06	4	2.14E+06	4	7.25E+05	7	1.18E+06	11	2.02E+05	33	1.06E+06	21	1.32E+06	1				
		ESTLPVGVYSIDDLKEYGR	40.45	6.65E+05	4	1.28E+05	15	9.08E+05	4	4.18E+05	15	4.65E+06	4	2.30E+06	2	7.47E+05	2	1.11E+06	16	1.46E+05	36	7.07E+05	9	1.17E+06	8				
		VHHVVQESPAGFLK	27.38	8.15E+05	3	1.71E+05	11	5.50E+05	50	6.36E+05	5	3.75E+06	6	1.60E+06	11	6.57E+05	11	1.14E+06	20	1.81E+05	38	6.02E+05	27	1.01E+06	2				
		ENDFLTFDAMR	41.45	1.07E+06	1	1.81E+05	11	1.10E+06	4	6.27E+05	7	7.47E+06	2	3.37E+06	2	1.16E+06	4	1.73E+06	13	3.23E+05	21	6.75E+05	8	1.60E+06	1				
Hay	B7Z0G1	SEGEDFIQGTLDGK	34.02	2.84E+05	10	8.34E+04	22	5.12E+05	10	2.16E+05	9	1.72E+05	6	2.59E+05	5	2.01E+05	8	3.09E+05	19	9.07E+04	36	1.45E+05	42	4.94E+04	28				
Mrn	Q9VUR1	NGTDAEAISPDAVR	24.41	4.58E+05	3	9.67E+04	13	4.57E+05	46	2.48E+05	5	1.46E+05	8	3.08E+05	17	2.44E+05	5	2.97E+05	15	1.07E+05	41	6.37E+04	50	1.91E+04	20				

[a]NCBI accession number as of Sept. 11, 2013

CV = coefficient of variation of three injections
